# Multi-environment testing for G×E interactions and identification of high-yielding, stable, medium-duration pigeonpea genotypes employing AMMI, GGE biplot, and YREM analyses

**DOI:** 10.3389/fpls.2024.1396826

**Published:** 2024-07-19

**Authors:** Naresh Bomma, H. B. Shruthi, Chandrakant D. Soregaon, Anil Gaddameedi, Krishnappa Suma, Jwala Pranati, Lohithaswa H. Chandappa, D. K. Patil, Niraj Kumar, S. Sandeep, Anilkumar Vemula, Prakash I. Gangashetty

**Affiliations:** ^1^ Pigeonpea Breeding, Accelerated Crop Improvement, International Crops Research Institute for the Semi-Arid Tropics, Hyderabad, Telangana, India; ^2^ College of Agriculture, Vijayapura, University of Agricultural Sciences (UAS), Dharwad, Karnataka, India; ^3^ Department of Genetics and Plant Breeding, Professor Jayashankar Telangana State Agricultural University, Hyderabad, Telangana, India; ^4^ Zonal Agricultural Research Station, University of Agricultural Sciences (UAS), Bengaluru, Karnataka, India; ^5^ Department of Plant Breeding, Agricultural Research Station, Badnapur, Maharashtra, India; ^6^ Department of Genetics and Plant Breeding, Birsa Agricultural University, Ranchi, Jharkhand, India; ^7^ Department of Genetics and Plant Breeding, Agricultural Research Station, Tandur, Telangana, India

**Keywords:** Genotype × environment interaction, AMMI, GGE biplot, ASV, YREM

## Abstract

Pigeonpea [*Cajanus cajan* (L.) Millspaugh] is a widely grown pulse with high seed protein content that contributes to food and nutritional security in the Indian subcontinent. The majority of pigeonpea varieties cultivated in India are of medium duration (<180 days to maturity), which makes it essential for breeders to focus on the development of stable high-yielding varieties. The diverse agroecological regime in the Indian subcontinent necessitates an efficient multi-environment study by taking into consideration genotype (G) × environment (E) interaction (GEI) that has a significant impact on traits like grain yield (GY) in developing high-yielding and widely adaptable varieties. In the present study, 37 pigeonpea genotypes were evaluated during the 2021 rainy season at ARS Badnapur, ARS Tandur, BAU Ranchi, GKVK Bengaluru, and ICRISAT Patancheru. The GEI was significant on the grain yield (*p* < 0.01), and hence, genotype + genotype × environment (GGE) and additive main effects and multiplicative interaction (AMMI) biplots along with AMMI stability value (ASV) and yield relative to environmental maximum (YREM) statistics were used to identify stable high-yielding genotypes. The interaction principal component analysis 1 and 2 (IPC1 and IPC2) explained 40.6% and 23.3% variations, respectively. Based on the rankings of genotypes, G37 (ICPL 20205), G35 (ICPL 20203), G8 (ICPL 19404), G17 (ICPL 19415), and G9 (ICPL 19405) were identified as ideal genotypes. Discriminativeness vs. representativeness identified GKVK Bengaluru as an ideal environment for comprehensive evaluation of test genotypes. However, ICPL 19405 was identified as the potentially stable high-yielding genotype for further testing and release across the test environments based on its mean grain yield (1,469.30 kg/ha), least ASV (3.82), and low yield stability index (YSI) of 13.

## Introduction

Pigeonpea [*Cajanus cajan* (L.) Millspaugh] is a grain legume crop of significant economic importance in developing countries in tropical and subtropical regions of the world (Varshney et al., 2012). Globally, pigeonpea occupies an area of 6.03 Mha, producing 5.3 MT with a productivity of 883.4 kg/ha. India contributes up to 65% of the global pigeonpea production with 4.3 MT produced in an area of 5.00 Mha with a productivity of 861.2 kg/ha (FAOSTAT, 2022). Cooking the dehulled split cotyledons of pigeonpea and serving them as a thick, spicy soup called dal with rice and bread is a common and traditional practice in India. Pigeonpea, containing 21%–25% protein (Saxena et al., 2010), is an ideal complement to cereals for a balanced diet. Beyond its nutritional value, pigeonpea contributes to sustainable agriculture through multiple uses, such as fertilizer (by fixing atmospheric nitrogen and aiding phosphorus release in soil), fuel, fodder, and pharmaceuticals (Mula and Saxena, 2010). Due to rising population demands, there has been an increased need for high pigeonpea seed production, leading to imports valued at nearly 116.57 million USD from African nations (Connect2India, 2021). Currently, the pigeonpea seed production chain constitutes varieties under four major maturity durations, namely, extra-early (90–120 days), early (121–150 days), mid-early (151–165 days), and medium (166–180 days). However, in India, the majority of the pigeonpea cultivated land is dominated by medium-duration maturity varieties. This necessitates the breeder to focus on the development of well-adapted and stable, high-yielding, medium-maturity varieties for the target environments.

The genotype (G) × environment (E) interaction (GEI) plays a significant role in determining the ability of a genotype to thrive in a given environment and ultimately decides its genetic ability to adapt and perform stably. Environment as a whole is a complex of multiple factors that largely include rainfall, temperatures, soil chemistry, soil humidity, disparities in soil type, and biotic stresses like disease and pests, which will together cause GEIs (Oladosu et al., 2016). In this context, extensive evaluation of genotypes across environments is necessary for plant breeding programs that are determined to develop widely adaptable varieties. Multi-environment trials offer the basis for assessing genotypic performance across environments and improving selection accuracy by taking into consideration GEI, which forms the primary factor in this investigation. A multitude of statistical models and tools have been developed to examine the impacts of GEI in mega-environment studies (Eberhart and Russell, 1966).

To facilitate the study of GEI, stability analysis makes use of a variety of statistics. Among them, analysis of variance (ANOVA) dissects the variation into genotype, environment, and genotype × environment effects. Deciphering the stability of the genotypes under study could be conducted by various univariate and multivariate statistical techniques. The best linear unbiased predictors (BLUPs) (Asfaw et al., 2022; Tena and Keneni, 2022; Mwale et al., 2023, 2023; Zhang et al., 2023) are estimated for the main and interaction effects of genotype and environment from the combined analysis of variance. It further allows the categorization of the best genotypes according to their environment-specific performance. Many studies have employed combined ANOVA for comparison of data based on multi-environment trials (Lee et al., 2023; Mwale et al., 2023). However, it limits the assessment of genotypic stability, as the model presumes that all genotypic variance and covariance of genotype pairs are the same. Thereby, it forms a restrictive variance–covariance structure of GEI and fails to understand the interaction of each genotype concerning the environments (Hu, 2014). In this direction, multivariate statistics like additive main effects and multiplicative interaction (AMMI) biplots, genotype + genotype × environment (GGE), and yield relative to environmental maximum (YREM) have been developed and employed successfully.

The primary models in GEI analysis are AMMI and GGE biplots (Alizadeh et al., 2017). The AMMI model combines principal component analysis (PCA) and ANOVA into a cohesive approach that may be applied to the analysis of multilocation trials (Zobel et al., 1988; Crossa et al., 1990; Gauch and Zobel, 1996). The GGE biplot uses a two-way table to graphically display a genotype × environment interaction (Yan et al., 2000). This tool is useful for a variety of tasks, including genotype evaluation (the mean performance and stability), test-environmental evaluation, and mega-environment analysis (e.g., “which-won-where” pattern), whereby particular genotypes can be recommended for specific areas (Ding et al., 2007). A unique kind of standardized estimate of a genotype’s performance with a negated environment primary effect is called YREM. Additionally, YREM provides an understandable measure of test genotype performance that is independent of genotype attendance (Yan, 1999), which aids in cultivar assessment and serves to identify crossover genotype × environment interaction (Ashwini et al., 2021; Spoorthi et al., 2021). Because each of the aforementioned statistics has merit, they are combined to produce more accurate results. The aim of the present study was therefore to identify the reliable medium-duration pigeonpea genotypes demonstrating stable higher yield using stability models such as AMMI biplot, GGE biplot, BLUP, and YREM.

## Materials and methods

### Genetic material

The experimental material comprised 37 pigeonpea genotypes of medium-maturity duration developed at ICRISAT, Patancheru, and three checks, viz., ICPL 87119, ICPL 8863, and a local check (high-performing variety of that environment). The list of the 37 genotypes used in the study is provided in **Supplementary Table 1**.

### Field trial evaluation

The genotypes along with three checks were evaluated in an alpha-lattice experimental design with three replications during the 2021 rainy across five distinct pigeonpea-growing regions in India, viz., ARS Badnapur (Maharashtra), ARS Tandur (Telangana), BAU Ranchi (Jharkhand), GKVK Bengaluru (Karnataka), and ICRISAT Patancheru (Telangana). The latitude and longitude of the test locations are provided in **Supplementary Table 2**. Each entry was sown in four rows of 4-m length with a row-to-row spacing of 75 cm and plant-to-plant spacing of 25 cm. The recommended package of practices was followed to raise a healthy crop. Data were recorded on five plants per plot, and observations were taken on five traits, namely, days to 50% flowering (DF) in days, days to maturity (DM) in days, plant height (PH) in cm, grain yield (GY) in kg/ha, and 100 seed weight (HSW) in g.

### Statistical analysis

Data on five quantitative traits were subjected to combined ANOVA across five environments to assess the main and interaction effects of genotypes and environments, considering genotypes, environments, replication, and block as random effects. The individual variance of environments was estimated and modeled to error distribution using the residual maximum likelihood (REML) estimator procedure with ASREMLv4.2 (Butler et al., 2017). BLUPs were estimated for all main and interaction effects from the combined analysis of variance.

Statistical analysis on AMMI and GGE to create the biplots was carried out in R studio version R 4.1.3 (Ahmad et al., 2023). Stability parameters such as AMMI stability value (ASV) and stability index (SI) were estimated based on the AMMI model to assess the relative stability of genotypes using R v4.1.3 (Chambers, 2008). YREM estimates in the present study were calculated based on BLUP values using Microsoft Excel software.

## Results

### Combined analysis of variance

Analysis of variance described the fixed and random effects of genotype, environment, and genotype × environment, and the results displayed a significant consequence of environment over the genotypes. Pooled ANOVA revealed that the main and interaction effects of genotype and environment were significant for all the traits in the study including days to 50% flowering (days), days to maturity (days), plant height (cm), 100 seed weight (g), and grain yield (kg/ha) (**Table 1**). Combined analysis of variance results revealed that the random effects of the environment, genotype, and genotype × environment interaction variance components are statistically significant from zero (*p* < 0.05). Moving further, the percent contribution from each component toward individual traits dissected through the variance components revealed that the environment contributed the highest variation for traits DF, DM, and PH of approximately 42.01%, 78.34%, and 55.52%, respectively. However, genotype contribution is more for HSW (44.10%), and genotype × environment contributed the highest variation for the GY of approximately 61.42%. With heritability being the key genetic component under selection, among the five traits studied, high heritability was observed for DM (85.91%) followed by DF (84.59%) and HSW (81.02%) (**Supplementary Table 3**). Correlation between the traits studied showed that DF had a high positive correlation with DM (r = 0.988, *p* < 0.01) and PH (r = 0.521, *p* < 0.01). Similarly, DM showed a positive correlation with PH (r = 0.513, *p* < 0.001) (**Supplementary Table 4**).

**Table 1:** Pooled analysis of variance of the 37 pigeonpea genotypes across five environments.

Random effect					
Effect	DF (days)	DM (days)	PH (cm)	HSW (g)	GY (kg/ha)
Environment	58.09 **	306.66 **	193.29 **	0.32 **	38,006.58 **
	**(42.01)**	**(78.34)**	**(55.52)**	(19.88)	(32.01)
Replication (Environment)	0.02	0.25 *	12.87 **	0.03	1,797.82
Block (Replication × Environment)	0	0.02	7.22 **	0	1,489.5 *
Genotype	39.21 **	43.94 **	28.83 **	0.71 **	7,797.37 *
	(28.35)	(11.22)	(8.28)	**(44.10)**	(6.57)
Genotype × Environment	40.99 **	40.87 **	126.03 **	0.58 **	72,910.51 **
	(29.64)	(10.44)	(36.20)	(36.02)	**(61.42)**
Residuals					
ARS Badnapur	2.46	2.63	70.4	–	8,832.15
ARS Tandur	2.29	2.28	23.2	0.07	25,651.56
BAU Ranchi	1.77	4.76	200.75	0.62	38,957.49
GKVK Bengaluru	5.99	6.06	150.09	1.83	10,386.6
ICRISAT Patancheru	9.32	13.29	66.6	0.47	60,715.06

* and ** are significant at the probability of 0.05 and 0.01, respectively. The percentage contribution of main and interaction effects of environment and genotype is in parentheses. Numbers highlighted in bold are the highest contributions.

DF, days to 50% flowering; DM, days to maturity; PH, plant height; HSW, 100 seed weight; GY, grain yield.

### AMMI biplots for assessing stability

Among different AMMI models, widely used AMMI 1 and AMMI 2 were determined in the present study to identify the highly adaptable and stable genotype through multi-environmental trial evaluation considering the genotype main effect and genotype × environment interaction effect. The results of AMMI 1 and AMMI 2 are presented below.

### Additive main effects and multiplicative interaction 1 biplot

**Figure 1:**
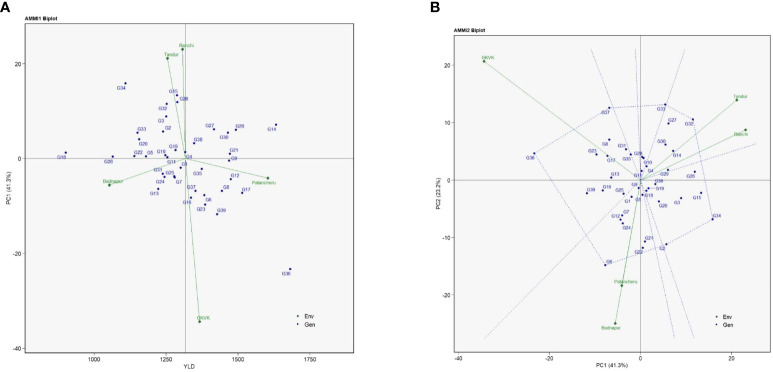
Additive main effects and multiplicative interaction (AMMI) biplots of test genotypes for grain yield (kg/ha). **(A)** AMMI 1 biplot. **(B)** AMMI 2 biplot. Badnapur, ARS Badnapur; Tandur, ARS Tandur; Ranchi, BAU Ranchi; Bengaluru, GKVK Bengaluru; Patancheru, ICRISAT Patancheru.

The AMMI 1 biplot links the variance of genotypes with the environmental effects, aiding in setting apart stable genotypes and selecting the best environment. AMMI 1 depicts the interactive principal component score (IPC1) against the mean of grain yield for both environments and the genotypes. The IPC1 score for grain yield was observed to be 41.3% (**Figure 1A**). Genotypes G11 (ICPL 19407), G9 (ICPL 19405), G5 (ICPL 19401), G22 (ICPL 19420), and G28 (ICPL 19426) were closer to the origin (zero IPC1 score), indicating that they recorded almost zero scores on the first IPC1. In contrast, genotypes such as G36 (ICPL 20204), G34 (ICPL 20202), and G15 (ICPL 19412) were far from the origin. Furthermore, the mean performance of environments or genotypes in AMMI 1 falling on the same parallel line about the ordinate was similar. The genotypes placed on the right side (first and fourth coordinates) of the biplot exhibited higher yield, among which the highest ones were G36 (ICPL 20204), G14 (ICPL 19411), G17 (ICPL 19415), and G29 (ICPL 19427). However, the genotypes placed on the left side of the center of the axis exhibited poor yield, among which the poorest ones were G18 (ICPL 19416), G28 (ICPL 19426), G22 (ICPL 19420), G34 (ICPL 20202), G33 (ICPL 20201), G20 (ICPL 19418), and G13 (ICPL 19410).

### Additive main effects and multiplicative interaction 2 biplot

AMMI 2 biplot of pigeonpea genotypes is illustrated in **Figure 1B**. AMMI 2 denotes the genotype × environment interaction to understand the response of genotypes in each test environment. The first two IPCs (IPC1 and IPC2) explained 64.5% (41.3% and 23.2%, respectively) of the total variation. Genotypes G6 (ICPL 19402), G32 (ICPL 19432), G33 (ICPL 20201), G34 (ICPL 20202), G36 (ICPL 20204), G37 (ICPL 20205), and G2 (ICPL 19395) were placed more distant from the origin and occupied the vertices of the polygon. Environment GKVK Bengaluru had the highest score on the IPC1 followed by ARS Badnapur, ICRISAT Patancheru, and ARS Tandur. However, the relatively lowest environmental score was observed for BAU Ranchi. For the grain yield, it was observed that genotypes G32 (ICPL 19432), G27 (ICPL 19425), G30 (ICPL 19428), and G14 (ICPL 19411) specifically adapted to ARS Tandur and BAU Ranchi, whereas G37 (CPL 20205), G36 (ICPL 20204) to GKVK Bengaluru, and G6 (ICPL 19402) specifically adapted to ARS Badnapur and ICRISAT Patancheru. It was determined using AMMI 2 that genotypes G11 (ICPL 19407), G9 (ICPL 19405), and G19 (ICPL 19417) were grouped in proximity to the origin.

### AMMI stability value, stability index, and yield stability index

The genotypes selected based on stability were further analyzed using the AMMI stability value. The ASVs in the present study were estimated using both interactive principal components, IPC1 and IPC2 scores, and it turns out that they significantly contributed to the total genotype × environment variance of grain yield. The ASV estimates are indirectly proportional to the stability of a genotype; i.e., the lower the estimate, the greater the stability. In the present study, the ASV among test genotypes ranged from 1.08 for G11 (ICPL 19407) to 40.72 for G36 (ICPL 20204). Genotypes G9 (3.82), G35 (6.48), G12 (9.26), and G21 (9.27) had the lowest ASVs along with the above trial mean grain yield performance (**Table 2**). Furthermore, the SI estimated for test genotypes across environments ranged from very low (13%) for G9 (ICPL 19405) to high (75%) for G34 (ICPL 20202). Among all the best three genotypes with the lowest SI values, G9 (13%) and G35 (24%) had the lowest ASVs as well and performed higher than the trial mean for grain yield. The YSI was determined based on the rank of the mean grain yield of genotypes (RY) and the rank of the AMMI stability value (RASV) for test genotypes, and it was observed that the lowest YSI was observed for G9 (ICPL 19405) of approximately 13 and the highest for G34 (ICPL 20202) of approximately 75. The best genotypes identified based on the mean grain yield, ASVs, and YSI are presented in **Table 2**.

**Table 2:** Estimates of mean grain yield and additive main effects and multiplicative interaction (AMMI) model-based parameters to assess the stability of the pigeonpea genotypes for grain yield.

Genotype code	Genotype name	Mean grain yield	RY	ASV	RASV	YSI	SI %
G1	ICPL 19394	1,293.89	22	4.36	3	25	27
G2	ICPL 19395	1,240.18	30	13.83	26	56	56
G3	ICPL 19396	1,258.83	25	14.59	29	54	54
G4	ICPL 19399	1,316.64	18	7.39	12	30	30
G5	ICPL 19401	1,180.82	33	7.06	11	44	44
G6	ICPL 19402	1,383.59	13	19.75	33	46	46
G7	ICPL 19403	1,280.90	24	10.14	19	43	43
G8	ICPL 19404	1,444.34	10	14.39	28	38	38
G9	**ICPL 19405**	**1,469.30**	9	**3.82**	4	**13**	13
G10	ICPL 19406	1,247.35	28	6.35	7	35	35
G11	ICPL 19407	1,254.65	26	1.08	1	**27**	27
G12	**ICPL 19408**	**1,475.25**	7	**9.26**	15	**22**	22
G13	ICPL 19410	1,223.30	32	11.55	22	54	54
G14	ICPL 19411	1,632.32	3	14.12	25	28	28
G15	ICPL 19412	1,308.61	19	22.36	35	54	54
G16	ICPL 19414	1,337.18	17	14.09	27	44	44
G17	ICPL 19415	1,514.81	4	13.59	22	26	28
G18	ICPL 19416	901.75	39	2.44	3	42	42
G19	ICPL 19417	1,291.46	22	4.25	5	**27**	27
G20	ICPL 19418	1,156.59	34	7.44	13	47	47
G21	**ICPL 19419**	**1,471.94**	8	**9.27**	15	**23**	25
G22	ICPL 19420	1,140.52	35	10.32	20	55	55
G23	ICPL 19421	1,385.97	12	17.31	32	44	44
G24	ICPL 19422	1,244.44	29	6.75	10	39	39
G25	ICPL 19423	1,300.74	21	7.67	14	35	35
G26	ICPL 19424	1,648.23	2	20.70	34	36	36
G27	ICPL 19425	1,416.40	11	12.87	23	34	34
G28	ICPL 19426	1,069.83	38	1.82	2	40	40
G29	ICPL 19427	1,492.78	5	11.60	19	24	26
G30	ICPL 19428	1,465.70	10	10.41	20	30	30
G31	ICPL 19430	1,238.53	31	**8.75**	15	46	46
G32	ICPL 19432	1,252.71	27	23.21	36	63	63
G33	ICPL 20201	1,139.20	36	17.08	31	67	67
G34	ICPL 20202	1,109.19	37	29.17	38	75	75
G35	**ICPL 20203**	**1,373.92**	15	**6.48**	4	**19**	24
G36	ICPL 20204	1,681.69	1	40.72	39	40	40
G37	ICPL 20205	1,352.19	17	14.77	30	47	47
**Checks**							
G38	ICPL 87119	1,476.67	6	26.03	38	44	44
G39	ICPL 8863	1,354.38	16	6.86	10	26	26

Genotypes with bold letters indicate the best genotypes with the above trial mean grain yield having ASV less than 10 and lowest YSI.

RY, rank of the test genotype based on mean grain yield; ASV, AMMI stability value; RASV, rank of the test genotype based on ASV; SI, stability index; YSI, yield stability index.

### Genotype × environment mean interaction plot

**Figure 2:**
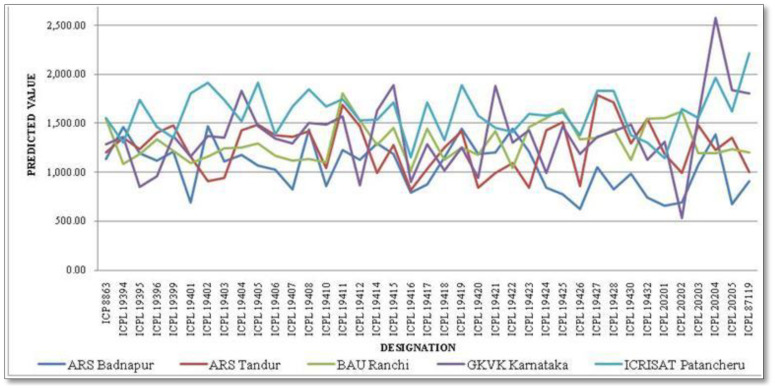
Interaction plot depicting the predicted value of 37 pigeonpea genotypes across environments.

A combined analysis of variance was used to calculate the genotype × environment means (BLUPs). The mean interaction plot (**Figure 2**) depicts how the genotype grain yield means were changed in their magnitude across the test environments. Out of 39 genotypes (including checks), 18 were performed above the trial mean wherein G36 (ICPL 20204) performed the highest with 1,441.26 kg/ha followed by G14 (ICPL 19411) with 1,419.43 kg/ha and G17 (ICPL 19415) with 1,383.97 kg/ha. The 10 best-performing pigeonpea genotypes for mean grain yield are enlisted in **Table 3**. In contrast, 21 genotypes performed below the trial mean with the lowest of 1,186.47 kg/ha by G18 (ICPL 19416). ICRISAT Patancheru recorded the highest mean value of 1,590.70 kg/ha, and the overall trial mean across the genotypes and environments was 1,318.88 kg/ha.

**Table 3:** Estimates of genotype, environment, and genotype × environment BLUP values of the 10 best genotypes for grain yield in pigeonpea.

EnvironmentGenotype	ARS Badnapur	ARS Tandur	BAU Ranchi	GKVK Bengaluru	ICRISAT Patancheru	Genotype mean
ICPL 20204	1,388.14	1,228.67	1,194.21	2,572.77	1,966.85	1,441.26
ICPL 19411	1,232.28	1,689.67	1,804.66	1,568.00	1,742.70	1,419.43
ICPL 19415	1,188.10	1,280.81	1,453.48	1,892.46	1,713.61	1,383.97
ICPL 19427	1,053.74	1,787.78	1,358.17	1,362.03	1,830.50	1,374.47
ICPL 19408	1,439.37	1,420.83	1,134.09	1,504.82	1,842.93	1,370.98
ICPL 19419	881.41	1,038.09	1,449.35	1,290.97	1,714.91	1,365.98
ICPL 19405	1,067.48	1,488.21	1,298.25	1,475.04	1,912.36	1,363.96
ICPL 19428	828.82	1,716.25	1,438.63	1,417.57	1,833.68	1,363.52
ICPL 19404	1,183.10	1,429.58	1,258.23	1,826.15	1,518.51	1,362.16
ICPL 19425	775.76	1,514.05	1,647.22	1,474.62	1,609.69	1,348.63
**Environmental mean**	1,070.55	1,259.19	1,308.84	1,365.12	1,590.70	

BLUP, best linear unbiased predictor.

### Genotype + genotype × environment biplot

**Figure 3:**
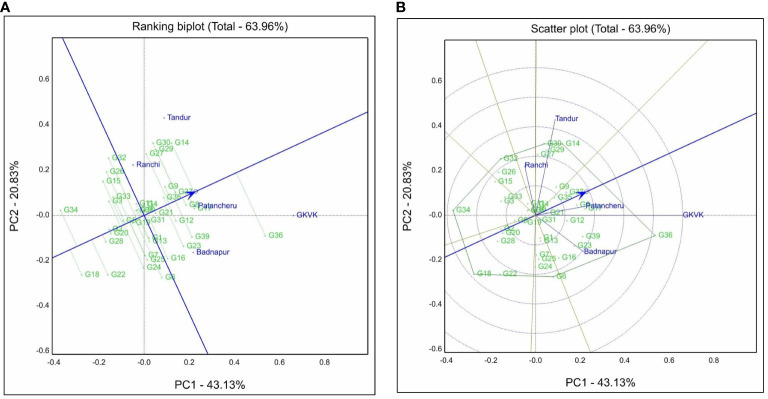
Genotype + genotype × environment (GGE) biplots for grain yield. **(A)** Ranking biplot shows best-performing genotypes and stability based on environment-focused scaling. **(B)** Polygon view of GGE biplot based on the symmetrical scaling for “which-won-where” pattern of genotypes and environments. Badnapur, ARS Badnapur; Tandur, ARS Tandur; Ranchi, BAU Ranchi; Bengaluru, GKVK Bengaluru; Patancheru, ICRISAT Patancheru.

The genotype + genotype × environment interaction biplot for the grain yield trait is represented in **Figure 3**. Under this, principal component 1 (PC1) contributed 43.13% and principal component 2 (PC2) contributed 20.83% to the total variation. In addition, PC1 and PC2 together contributed approximately 63.96% to the total variation for grain yield. GGE biplot facilitates visual cultivar evaluation and assesses the relative stability of test genotypes. A ranking biplot drawn thus classifies the genotypes based on the environments. Genotypes located near the IPC axis and around the Average environment coordinate (AEC) point are the ideal genotypes. G35 (ICPL 20203), G37 (ICPL 20205), G11 (ICPL 19407), and G9 (ICPL 19405) genotypes had shorter projections from the AEC axis across environments. Meanwhile, G18 (ICPL 19416), G20 (ICPL 19418), and G28 (ICPL 19426) had shorter projections but were placed far away from the AEC arrow (**Figure 3A**).

A polygon is constructed by connecting all the farthest genotypes in the biplot wherein genotypes G36 (ICPL 20204), G6 (ICPL 19402), G18 (ICPL 19416), G34 (ICPL 20202), G32 (ICPL 19432), G30 (ICPL 19428), and G14 (ICPL 19411) occupied the corners of the polygon. Genotype G6 (ICPL 19402) was placed near the environment ARS Badnapur, and G14 (ICPL 19411) and G30 (ICPL 19428) were closely placed near the environmental vector of BAU Ranchi. For the ARS Tandur environment, the genotypes in the vicinity were G14 (ICPL 19411) and G30 (ICPL 19428). Genotype G36 (ICPL 20204) was situated near both GKVK Bengaluru and ICRISAT Patancheru environmental vectors.

Environmental vectors were represented on the biplot by perpendicular lines from the origin. The environmental vector of GKVK Bengaluru was the longest as compared to that of other environments. Biplot was divided into mega-environments by lines originating from the biplot origin, based on grain yield data across environments. Test environments were divided into two mega-environments. BAU Ranchi and ARS Tandur were placed in one mega-environment, while ICRISAT Patancheru, ARS Badnapur, and GKVK Bengaluru were placed in another (within two sectors). Few genotypes occupying the corners of the polygon fall in the sector without any environmental vectors in it, such as ICPL 19416, ICPL 20202, and ICPL 19432. Genotype G36 (ICPL 20204) had a longer projection length from the axis and occupied the vertex of the polygon in the first mega-environment (**Figure 3B**). In contrast, genotypes G30 (ICPL 19428) and G14 (ICPL 19411) were placed near the environmental vectors of BAU Ranchi and ARS Tandur. The angle between environmental vectors talks about the correlation of results among them. BAU Ranchi and ARS Badnapur vectors formed an obtuse angle (angle > 90°) between them, indicating that the results of the two environmental vectors are negatively correlated.

### Estimation of yield relative to environmental maximum model for cultivar assessment

**Figure 4:**
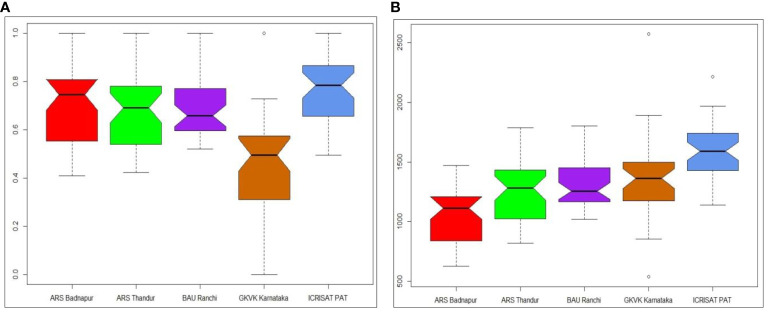
Box–whisker plots depicting **(A)** yield relative to environmental maximum (YREM) values of 37 pigeonpea genotypes across test environments and **(B)** variation in predicted values of genotypes for test environment.

In the present investigation, variation in YREM was evident in each of the environments and was significantly different from others. Variations in YREM and predicted values across environments are depicted in **Figure 4**. The variation in the estimates was lower in BAU Ranchi, and genotypes showed more variation in ARS Badnapur. The whisker plot of GKVK Bengaluru with lower YREM values occupied a lower part of the graph than other environments. YREM estimates of best pigeonpea genotypes for grain yield across environments are presented in **Table 4**. Genotypes G14 (ICPL 19411) and G36 (ICPL 20204) both had the highest average YREM value of 0.84 estimated across five environments. Subsequently, genotypes G21 (ICPL 19419), G29 (ICPL 19427), G17 (ICPL 19415), and G12 (ICPL 19408) showed YREM values of 0.77 each, whereas G9 (ICPL 19405) and G30 (ICPL 19428) showed YREM values of 0.75 each.

**Table 4:** Estimates of yield relative to environmental maximum (YREM) of 10 best pigeonpea genotypes across environments for grain yield.

Genotype	ARS Badnapur	ARS Tandur	BAU Ranchi	GKVK Bengaluru	ICRISAT Patancheru	Average YREM
ICPL 19411	0.82	0.94	1	0.59	0.87	0.84
ICPL 20204	0.93	0.66	0.61	1	1	0.84
ICPL 19419	0.99	0.78	0.65	0.48	0.97	0.77
ICPL 19427	0.7	1	0.71	0.51	0.93	0.77
ICPL 19415	0.79	0.69	0.77	0.73	0.86	0.77
ICPL 19408	0.96	0.78	0.58	0.58	0.94	0.77
ICPL 19405	0.72	0.82	0.68	0.56	0.99	0.75
ICPL 19428	0.54	0.95	0.77	0.54	0.93	0.75
ICPL 19404	0.78	0.78	0.66	0.71	0.73	0.73
ICPL 19402	1	0.47	0.61	0.53	0.99	0.72
ICPL 19425	0.51	0.83	0.91	0.56	0.79	0.72
ICPL 19421	0.82	0.52	0.77	0.72	0.7	0.7

## Discussion

Enhancing the productivity of pigeonpea assumes specific significance in the Indian subcontinent to meet the increasing demand and nutritional security in terms of the daily protein requirement of the predominantly vegetarian population who consume pigeonpea in the form of dry split dal. The genotype × environment interactions, or multi-environment trials, are essential components of crop genetic improvement and breeding programs. According to the combined analysis of variance, the percentage contribution of both environment and genotype × environment interaction effects explained more than 70% of the variation for all traits except HSW, indicating that the individual influence of genotypes did not differ significantly and that there is significant non-crossover interaction that exists (G×E); that is, the genotype mean performance will be affected by the environments. Consequently, the current study uses multi-environmental trials to establish a few stability models to find stable and adaptable genotypes and also to evaluate crossover interactions, such as AMMI, GGE, and YREM.

### Additive main effects and multiplicative interaction 1 biplot

AMMI is one of the essential models for assessing the impact of genotype × environment interactions on economically important traits like grain yield and its related traits across several environments. Understanding the interplay between genotypes and the relevant environments is made possible by the AMMI model. AMMI 1 is primarily used to discover high potential yield and stability, according to Olivoto et al. (2019). While analyzing AMMI 1, Kılıç (2014) reported that the genotype and environment mean when positioned parallel to the ordinate indicates nearly equal performance. However, a higher yield is displayed by the genotypes positioned on the right side of the biplot’s center than by those on the left. These results were found similar to our findings for grain yield. The degree of interaction between genotypes increases, which differs from the origin; the less interactive the genotypes, the closer they are to the origin. In the present study also, genotypes placed closer to the origin were less interactive, suggesting that the genotypes exhibit significant adaptability and possess favorable characteristics for achieving grain yield, whereas genotypes like G36 (ICPL 20204), which was placed farther from the origin, were found to be more interactive, suggesting that these genotypes demonstrated a constrained capacity for adaptation and are better suited for environments characterized by limited conditions.

Within the framework of the AMMI 1 study, it has been noted that genotypes close to the IPC1 axis center region show higher stability with fewer interaction effects. These genotypes exhibit a broad spectrum of adaptation to diverse environmental conditions as a result. In contrast, when the environment and genotype on the IPC axis have congruent polarity, a positive interaction is detected. However, when the environment and genotype have different polarities, an undesirable interaction results. The results obtained from our investigation align with the recorded findings (Kaya et al., 2002; Muniswamy et al., 2021; Reddy et al., 2023; Kona et al., 2024). The results of this study provide empirical support for the implementation of AMMI 2, showing that the AMMI model demonstrates a satisfactory degree of consistency with the gathered data.

### Additive main effects and multiplicative interaction 2 biplot

The environmental and genotypic scores of the first two AMMI components are used to create the AMMI 2 biplot. Understanding the role of genotype × environment interaction and the adaptability of the genotypes in the test environments is aided by the IPC1 and IPC2 scores. The first two principal components explained approximately 64.5% of the variation. From the AMMI ANOVA (**Supplementary Table 5**), the first four IPCs [IPC1 (41.23%), IPC2 (23.20%), IPC3 (20.30%), and IPC4 (15.1%)] explained approximately 99.83% of the variation in genotype × environment interactions that were found to be significant. However, several other authors have also reported the predominance of IPC1 and IPC2 interaction components in explaining maximum variation for yield traits (Gauch and Zobel, 1996; Khan et al., 2021; Esan et al., 2023; Kona et al., 2024).

From the AMMI 2 biplot, more general adaptation is expressed by genotypes that are closer to the ordinate axis, and more environment-specific adaptability is expressed by genotypes that are farther from it. AMMI 2 was used in this investigation to identify the specific adaptable genotypes for the respective environments. The results of the current study are consistent with those of Purchase (1997), and also similar findings were reported by Kılıç (2014) and Khan et al. (2021). The environment GKVK Bengaluru exhibits longer vectors, suggesting their greater contribution toward genotype × environment interactions and hence more discriminatory ability of the genotypes evaluated. Genotypes G36 (ICPL 20204) and G37 (ICPL 20205) were found to be more adaptable to the highly discriminating environment of GKVK Bengaluru in the current study.

To quantify and rank genotypes according to their yield stability, the AMMI model does not include a quantitative stability measure. Purchase et al. (2000) developed the ASV measure as a solution to this issue. The genotype stability is indicated by the ASV. Low ASV genotypes are thought to be more stable, whereas high levels indicate less stable genotypes (Hagos and Abay, 2013). Selection is not necessary because a genotype that routinely produces low yields can nonetheless be stable in terms of yield performance (Yan and Tinker, 2006). The most stable genotypes do not always have the best yield performance in certain situations (Oliveira and Godoy, 2006). For this reason, when estimating the YSI, high grain yield is taken into account together with stability. To choose varieties, the YSI combines yield and stability in a variety of settings into a single index. The YSI sums the rank of mean yield across environments with the rank of the ASV of genotypes (Tumuhimbise et al., 2014; Baraki et al., 2014). Genotypes with lower YSI are desirable since they combine high mean yield performance with stability (Tumuhimbise et al., 2014; Baraki et al., 2014; Bose et al., 2014). Based on the YSI, genotypes G9 (ICPL 19405), G12 (ICPL 19408), G14 (ICPL 19411), G21 (ICPL 19419), G17 (ICPL 19415), G29 (ICPL 19427), and G35 (ICPL 20203) were selected as combining high yield performance with stability.

### Genotype + genotype × environment biplot

GGE biplot displays genotype + genotype × environment interaction study using site regression analysis, which explains genotype evaluation (stability and adaptability), environment evaluation (representativeness and discriminating power), and mega-environment (which-won-where) evaluation (Yan et al., 2000; Vemula and Parthasarathy, 2023). The first two principal components explained 64% of the total variation. Ideal genotypes are supposedly located near the IPC axis around the AEC point. The relative lengths of projections of the genotypes from AEC are indicative of their relative stability. The shorter the length of the projections of genotypes from AEC, the greater the stability of the genotypes and vice versa (Yan and Kang, 2003). Genotypes G35 (ICPL 20203), G37 (ICPL 20205), G11 (ICPL 19407), and G9 (ICPL 19405) had shorter projections from the AEC axis and were present around the AEC arrow (toward the AEC). These genotypes are widely adaptable, and performance is consistent across the environments. Genotypes G2 (ICPL 19395), G20 (ICPL 19418), and G28 (ICPL 19426) also had shorter projections but were placed in opposite directions of the AEC axis, indicating that they are highly stable and have poor performance of grain yield. Similarly, GGE biplots were also used by researchers to identify stable genotypes in previous studies in pigeonpea (Srivastava et al., 2012; Kumar et al., 2021; Rao et al., 2022).

According to the GGE biplot, the farthest genotypes occupying the vertex of the polygon are the specifically adaptable ones. Vertex genotypes such as G36 (ICPL 20204), G6 (ICPL 19402), G18 (ICPL 19416), G34 (ICPL 20202), G32 (ICPL 19432), G30 (ICPL 19428), and G14 (ICPL 19411) showed varied performance in different environments and were found to adaptable to specific environments. Among the vertex genotypes, G6 (ICPL 19402) was the high-performing genotype at ARS Badnapur. In the BAU Ranchi environment, G14 (ICPL 19411) and G30 (ICPL 19428) were well-suited. Meanwhile, G14 (ICPL 19411) and G30 (ICPL 19428) were well adapted to the ARS Tandur environment. For both GKVK Bengaluru and ICRISAT Patancheru, G36 (ICPL 20204) was recognized as the specifically adapted genotype. Genotypes G18 (ICPL 19416), G34 (ICPL 20202), and G32 (ICPL 19432) falling in the sectors without environmental vectors in it cannot be considered adaptable genotypes due to their poor performance across test environments (Khan et al., 2021; Rana et al., 2021; Ruswandi et al., 2022).

Each environment is represented as a vector in the biplot by drawing the perpendicular lines from the origin. Longer environmental vectors are indicative of their discriminative ability. GKVK Bengaluru environment showed high discriminating power depicting the variation among the genotypes as high in this environment. Genotypes express themselves, thereby providing a stage to discriminate them based on their performance. Perpendicular lines drawn from the origin divide the biplot into sectors called mega-environments (Kumar et al., 2021). A mega-environment is made up of a group of environments existing in between the sectors. If any of the genotypes fall in the mega-environment region, then they are considered as specific adaptable (Yan, 1999). BAU Ranchi and ARS Tandur shared similar conditions, while ICRISAT Patancheru, ARS Badnapur, and GKVK Bengaluru environments provided similar growing conditions to the experimental material. The angle formed between the environmental vectors talks about the crossover interaction. When the cosine angle between the environmental vectors is acute (<90°), the results from the two environments are correlated (Yan et al., 2000; Farshadfar et al., 2012), thereby indicating that genotype ranks are not changing across the environments. If the cosine angle between environmental vectors is obtuse (>90°), then genotype ranks are changing across the environments. In the current study, BAU Ranchi and ARS Badnapur environmental vectors formed an obtuse angle; then, the presence of crossover interaction between BAU Ranchi and ARS Badnapur (angle >90°) denoted that the genotype ranks were changing across these two environments.

### Yield relative to environmental maximum

To identify crossover genotype × environment interaction and measure the decline in test genotypes’ potential grain yield due to crossover genotype × environment interaction, YREM, a straightforward statistic, was employed (Spoorthi et al., 2021). The greater the genotype’s YREM value, the smaller the crossover genotype × environment interaction magnitude and the smaller the genotype’s potential drop in grain production even in the presence of crossover genotype × environment interaction. The performance of the best genotypes is what it can potentially attain in a particular environment. Therefore, YREM serves as a proxy for the crossover GEI’s magnitude. Consequently, the average YREM of a genotype evaluated across environments must equal 1.0 in the absence of crossover genotype × environment interaction. Any deviation of a genotype’s YREM from 1.0 is understood as a crossover genotype × environment interaction-related reduction in the genotype’s achievable grain yield (Yan, 1999). In the present investigation, YREM values varied across environments, indicating that yield reduction was noticed as the effect of genotype × environment interaction. Among all the test environments, in GKVK Bengaluru, lower YREM values were seen, which is due to the higher yield reduction. A higher YREM value of 0.84 was achieved by G14 (ICPL 19411) and G36 (ICPL 20204) across five environments, indicating that 84% of their grain yield potential can be realized across the test environments, making them relatively stable genotypes. The estimates of YREM were also employed earlier for diagnosing genotype × environment interaction in studies conducted by Yan (2000), Fikere et al. (2008), and Ashwini et al. (2021).

## Conclusion

Enhancing pigeonpea productivity is crucial for the Indian subcontinent, addressing increasing demand and nutritional needs, particularly for the predominantly vegetarian population. This study utilized multi-environment trials (AMMI, GGE, BLUP, and YREM) to identify medium-maturity genotypes that are adaptable and stable. Combined ANOVA for traits like DF, DM, PH, HSW, and GY indicated significant differences among 37 pigeonpea genotypes, revealing varied performances across environments. Significant genotype × environment interactions highlighted the necessity for in-depth analyses to refine cultivar selection. The environment contributed most to variations in DF, DM, and PH, while genotype × environment had the highest influence on GY. Genotype G36 (ICPL 20204) was identified as widely adaptable with high BLUP values. AMMI and GGE biplots further assessed the impact of genotype × environment interactions. AMMI 1 biplot analysis identified high-yield and stable genotypes, while AMMI 2 provided insights into adaptability using IPC analysis scores. The GGE biplot effectively ranked genotypes and identified ideal test environments. Genotypes G37 (ICPL 20205), G35 (ICPL 20203), G8 (ICPL 19404), G17 (ICPL 19415), and G9 (ICPL 19405) were found to be stable and high-yielding, while environments like GKVK Bengaluru demonstrated high discriminatory power. YREM values were used to quantify crossover genotype × environment interaction effects, with genotypes G14 (ICPL 19411) and G36 (ICPL 20204) showing minimal yield reduction, indicating stability. These comprehensive analyses support the selection of genotypes that combine high-yield performance with stability across diverse environments, aiding in the development of resilient pigeonpea cultivars.

## Data availability statement

The datasets presented in this study can be found in online repositories. The names of the repository/repositories and accession number(s) can be found in the article/**Supplementary Material**.

## Author contributions

NB: Conceptualization, Data curation, Investigation, Methodology, Writing – original draft, Writing – review & editing. HS: Conceptualization, Writing – review & editing. SC: Investigation, Writing – review & editing. AG: Methodology, Writing – original draft, Writing – review & editing. KS: Methodology, Writing – original draft, Writing – review & editing. JP: Data curation, Methodology, Writing – review & editing. LC: Investigation, Writing – review & editing. PD: Investigation, Writing – review & editing. NK: Investigation, Writing – review & editing. SS: Investigation, Writing – review & editing. AV: Data curation, Formal analysis, Methodology, Software, Visualization, Writing – review & editing. PG: Funding acquisition, Investigation, Methodology, Project administration, Resources, Supervision, Visualization, Writing – review & editing.
