# Supplementary material for: Multi-environment testing for G×E interactions and identification of high-yielding, stable, medium-duration pigeonpea genotypes employing AMMI, GGE biplot, and YREM analyses
Source: Front Plant Sci. 2024 Jul 19;15:1396826. doi: 10.3389/fpls.2024.1396826 (PMC11295248; doi:10.3389/fpls.2024.1396826)
Supplement: Supplementary file 1 [file Table_1.docx]

**Supplementary Table 1: List of pigeonpea genotypes used in the present study**

| **Sl. No.** | **Genotype** | **Sl. No.** | **Genotype** |
| --- | --- | --- | --- |
| 1 | ICPL 19394 | 21 | ICPL 19419 |
| 2 | ICPL 19395 | 22 | ICPL 19420 |
| 3 | ICPL 19396 | 23 | ICPL 19421 |
| 4 | ICPL 19399 | 24 | ICPL 19422 |
| 5 | ICPL 19401 | 25 | ICPL 19423 |
| 6 | ICPL 19402 | 26 | ICPL 19424 |
| 7 | ICPL 19403 | 27 | ICPL 19425 |
| 8 | ICPL 19404 | 28 | ICPL 19426 |
| 9 | ICPL 19405 | 29 | ICPL 19427 |
| 10 | ICPL 19406 | 30 | ICPL 19428 |
| 11 | ICPL 19407 | 31 | ICPL 19430 |
| 12 | ICPL 19408 | 32 | ICPL 19432 |
| 13 | ICPL 19410 | 33 | ICPL 20201 |
| 14 | ICPL 19411 | 34 | ICPL 20202 |
| 15 | ICPL 19412 | 35 | ICPL 20203 |
| 16 | ICPL 19414 | 36 | ICPL 20204 |
| 17 | ICPL 19415 | 37 | ICPL 20205 |
| 18 | ICPL 19416 | 38 | ICPL 87119 (C) |
| 19 | ICPL 19417 | 39 | ICPL 8863 (C) |
| 20 | ICPL 19418 | 40 | Local Check |

**Supplementary Table 2. Geographic coordinates for test environments in the present experiment**

| **Locations** | **Geographic coordinate** | |
| --- | --- | --- |
|  | **Latitude** | **Longitude** |
| GKVK Bengaluru | 13.08°N | 77.58°E |
| ARS Tandur | 17.01°N | 77.35°E |
| BAU Ranchi | 23.17°N | 85.19°E |
| ICRISAT Patancheru | 17.54°N | 78.28°E |
| ARS Badnapur | 19.87°N | 75.73°E |

**Supplementary Table 3. The heritability and coefficient of variation for days to 50% flowering (DF), Days to maturity (DM), Plant Height (PH), Seed weight (SW) and Grain yield (GY)**

| **Traits** | **H^2^** | **CV** |
| --- | --- | --- |
| **DF (days)** | 84.599 | 2.038 |
| **DM (days)** | 85.909 | 1.524 |
| **PH (cm)** | 52.034 | 5.81 |
| **HSW (g)** | 81.023 | 8.599 |
| **GY (kg ha^-1^)** | 32.079 | 12.89 |

**Supplementary Table 4. The correlation matrix among days to 50% flowering (DF), Days to maturity (DM), Plant height (PH), Seed weight (SW) and Grain yield (GY).**

| *\* | ***DF (days)*** | ***DM (days)*** | ***PH (cm)*** | ***HSW (g)*** | ***GY (Kg ha^-1^)*** |
| --- | --- | --- | --- | --- | --- |
| ***DF (days)*** | 1 |  |  |  |  |
| ***DM (days)*** | 0.988** | 1 |  |  |  |
| ***PH (cm)*** | 0.52** | 0.513** | 1 |  |  |
| ***HSW (g)*** | -0.023 | -0.077 | 0.242 | 1 |  |
| ***GY (Kg ha^-1^)*** | -0.094 | -0.114 | -0.207 | 0.031 | 1 |

**Supplementary Table 5. Analysis of variance component for Additive main effects and multiplicative interaction (AMMI) models.**

| **Source of variation** | **Df** | **SS** | **MSS** | **F value** | **Pr (>F)** |  | **% variation** |
| --- | --- | --- | --- | --- | --- | --- | --- |
| Genotype | 38 | 13224532 | 348014 | 11.58 | 9.35E-42 | ** |  |
| Environment | 4 | 18517920 | 4629480 | 24.23 | 2.31E-05 | ** |  |
| Rep (Env) | 10 | 1910390 | 191039 | 6.36 | 9.19E-08 | ** |  |
| Genotype*Environment | 148 | 35434240 | 233120 | 7.76 | 1.39E-56 | ** |  |
| PC1 | 41 | 14643642 | 357162 | 11.89 | 0 | ** | 41.23 |
| PC2 | 39 | 8230170 | 211030 | 7.02 | 0 | ** | 23.2 |
| PC3 | 37 | 7198794 | 194562 | 6.48 | 0 | ** | 20.3 |
| PC4 | 35 | 5361615 | 153189 | 5.1 | 0 | ** | 15.1 |
| Residuals | 362 | 10875928 | 30044 |  |  | ** |  |
| Total | 714 | 112472136 | 157524 |  |  |  |  |
